# Editorial: 7th international symposium on peripheral nerve regeneration: peripheral nerve regeneration - advances and new directions

**DOI:** 10.3389/fcell.2025.1701185

**Published:** 2025-09-18

**Authors:** Rui Alvites, Óscar Darío García

**Affiliations:** ^1^ Centre for the Studies of Animal Science, Institute of Agrarian and Agri-Food Sciences and Technologies, University of Oporto, Porto, Portugal; ^2^ Departamento de Clínicas Veterinárias, Instituto de Ciencias Biomedicas Abel Salazar, Universidade do Porto, Porto, Portugal; ^3^ Instituto Universitário de Ciências da Saúde, Cooperativa de Ensino Superior Politecnico e Universitario CRL, Gandra, Portugal; ^4^ Associate Laboratory for Animal and Veterinary Science (AL4AnimalS), Lisbon, Portugal; ^5^ Tissue Engineering Group, Department of Histology, University of Granada and Instituto de Investigación Biosanitaria ibs.GRANADA, Granada, Spain

**Keywords:** nerve regeneration, nerve repair, biomatenals, cell-based therapies, tissue engeneering, farmacological treatment, surgical procedures

Despite the remarkable advances achieved in recent years, peripheral nerve injury remains a complex medical challenge in the field of neurosciences, with severe physical and psychological consequences. The intrinsic capacity of peripheral nerves to regenerate is well described in the literature, but this capacity is limited and often associated with inadequate structural reorganization, resulting in a limited motor and sensory recovery and in general poor prognosis. Therefore, several research areas are attempting to develop new, innovative therapies to promote peripheral nerve regeneration, and in many of them, progress has been remarkable.

The Research Topic “Peripheral Nerve Regeneration - Advances and New Directions,” created within the context of the seventh International Symposium on Peripheral Nerve Regeneration, held in Milan from May 7th to 9th, 2024, brought together a Research Topic of high-quality research and review articles that encompass recent relevant advances in peripheral nerve regeneration after injury, both in a basic and translational research approach. The authors of the published works, both event participants and external ones, explored the topic of peripheral nerve regeneration in fields as diverse as cell-based therapies, biomaterials, tissue engineering, pharmacological treatments, nutritional therapies and new surgical approaches, demonstrating the variability of research fields currently dedicated to this issue.

In the field of biomaterials and tissue engineering, Deininger et al. offer a critical mini-review of biomaterials for traumatic nerve injuries, analyzing the advantages and limitations of hydrogels, conduits, and functional scaffolds. They highlight considerations regarding biocompatibility, degradation rate, and the incorporation of trophic factors. The work serves as a practical reference for researchers designing conduit devices with clinical potential. Stocco et al. provide a comprehensive review of strategies for modeling the microenvironment in conduits using self-assembling peptides (SAPs), evaluating physicochemical properties, functionalization with mimetic epitopes and results in preclinical models. The review highlights SAPs as a versatile platform, although it highlights challenges of scale, manufacturing, and regulatory certification for clinical translation. Gregory et al. report a nanofibrillar scaffold produced by emulsion electrospinning and loaded with GDNF, demonstrating controlled release and favorable effects on axonal elongation in experimental models. The study provides proof of concept for topography-oriented local trophic factor delivery systems, proposing pathways to optimize release profiles and vascular integration. Redolfi Riva et al. revisit the synergy between external stimuli (electrical and mechanical stimulation, phototherapy) and nanostructured biomimetic scaffolds, emphasizing that the confluence of physical and biochemical cues improves axonal guidance, Schwann maturation, and vascularization. The synthesis identifies gaps in experimental protocols and proposes standardization of physical parameters to improve translational reproducibility.

Exploring new pharmacological treatments, Alhamdi et al. compile evidence on pharmacological modulators capable of reprogramming Schwann cells to the “repair” phenotype, including pathways involving c-Jun, STAT3, IGF-1, and NRG1. The review discusses candidate drugs and delivery strategies, highlighting the critical role of pharmacology in preserving the repair phenotype during chronic denervation and its relevance in combination with biomaterial approaches. Fischer presents a perspective on the parthenolide-like cnicin, discussing its pro-regenerative mechanisms and proposing it as a pharmacological candidate to enhance axonal growth. The author argues for the need for additional mechanistic studies and *in vivo* validation. Hazer Rosberg et al. describe the effects of the lactoferrin-derived peptide PXL01 on nerve regeneration in sciatic nerve reconstruction models, including diabetic variants, demonstrating an influence on axonal growth and partial functional recovery. The work highlights the translational potential of matrix/inflammation-modulating peptides as adjuvants for nerve reconstruction, highlighting the need for pharmacokinetic and dose-ranging studies in larger preclinical models before clinical trials.

For new surgical approaches, Tratnig-Frankl et al. compare degenerated and fresh nerve grafts in the reconstruction of critical defects and demonstrate comparable quality in structural and functional parameters, which has practical implications for tissue banking and surgical logistics, namely, reducing the need for fresh tissue and increasing therapeutic options. The discussion includes recommendations on processing conditions and evaluation criteria. Harnoncourt et al. analyze axonal regeneration and innervation ratio after end-to-side supercharged nerve transfer, providing morphological and functional data that support this technique as a strategy to optimize innervation ratio and accelerate motor reinnervation. The study discusses surgical implications and donor/recipient selection criteria, highlighting the need for standardization of functional measures for multicenter comparisons.

In the field of cell-based therapies, Alvites et al. describe a translational study in sheep where the use of a conditioned medium produced from mesenchymal stem cells of the olfactory mucosa, when combined with neural guide tubes, improves tissue organization and morphometric parameters of nerve regeneration. The large-animal model strengthens the preclinical evidence and supports the safety/tolerability assessment of the developed therapeutic combination prior to clinical trials. Lopes et al. present an *in vitro* characterization of dental pulp stem cells (DPSCs) and their secretome, evaluating biomarkers, secretory profile, and compatibility with nerve guides, providing robust experimental foundations for future *in vivo* applications. The authors emphasize that the use of secretome and conditioned media can mitigate risks associated with direct cell transplantation and constitute a practical alternative for translational therapies.

In their article, Stenberg et al. analyzed DA.Vra1-congenic mice compared to the DA parental strain after sciatic nerve injury and repair. Although no differences were observed in initial axonal regeneration, increased Schwann cell apoptosis and higher expression of genes related to stress and apoptosis were observed. These results highlight the complexity of the genetic contribution to the nerve repair response and suggest that early molecular changes are not always reflected in immediate morphofunctional differences.

In studies that aim to help unravel the complex mechanisms associated with nerve injury and regeneration, García-Bejarano et al. investigated RGS16 expression in acute and chronic injuries, proposing a feedback mechanism where RGS16 may negatively regulate NRG1 release via modulation of GPCR/ErbB transactivation. The data add a new layer of understanding about temporal signaling in nerve degeneration/regeneration and suggests RGS16 as a possible biomarker or modulatory target. Jablonka-Shariff et al. demonstrate that the absence of the transcription factor T-bet limits neuromuscular junction recovery after injury in knockout models, implicating T-bet in Schwann cell terminal signaling and local immune modulation. The study thus links immunoregulatory factors to motor outcomes and suggests targeted exploration of immune-neural pathways to improve reinnervation.

In his opinion article, Nardini explore the importance of considering neuroimmune modulators in chronic healing processes, highlighting interactions between nerves, immunity, and the extracellular matrix that influence both skin repair and associated nerve recovery. The article suggests lines of translational research that integrate neuroimmune therapy to improve outcomes in chronic injuries and adverse biomechanical environments.

Finally, Ronchi et al. present evidence that the gut microbiota influences the maturation and myelination pattern of optic nerve fibers in murine models, raising the hypothesis of a gut-eye/nerve axis that can modulate plasticity and response to injury. This work broadens the perspective of systemic factors that influence regeneration and opens avenues for nutritional or probiotic interventions as adjuvants.

This Special Research Topic comprises 16 articles, including 10 original articles, 5 reviews, and 1 opinion piece. Among the original articles, seven *in vivo* articles are particularly noteworthy, as they contribute to the clinical translation of new therapies for regenerating peripheral nerve injuries. This compilation of articles represents the most innovative achievements in the field of peripheral nerve regeneration, highlighting the significant advances made in understanding the intricate mechanisms of nerve injury and repair, as well as the promise of new therapies that could be revolutionary in the near future. The Editors would like to extend their special thanks to all the authors who collaborated with them and participated in this editorial project.

